# Pathogen spectrum and microbiome in lower respiratory tract of patients with different pulmonary diseases based on metagenomic next-generation sequencing

**DOI:** 10.3389/fcimb.2024.1320831

**Published:** 2024-10-31

**Authors:** Rujun Hong, Sheng Lin, Siting Zhang, Yaxing Yi, Lanfeng Li, Haitao Yang, Zhenshan Du, Xuefang Cao, Wenjie Wu, Ruotong Ren, Xiujuan Yao, Baosong Xie

**Affiliations:** ^1^ Department of Pulmonary and Critical Care Medicine, Fujian Provincial Hospital, Fujian Provincial Clinical Medical College, Fujian Medical University, Fuzhou, China; ^2^ Medical Department, Matridx Biotechnology Co., Ltd, Hangzhou, China; ^3^ Institute of Biophysics, Chinese Academy of Sciences, Beijing, China

**Keywords:** pulmonary disease, lower respiratory tract, mNGS, pathogen, microbiome

## Abstract

**Introduction:**

The homeostasis of the microbiome in lower respiratory tract is crucial in sustaining normal physiological functions of the lung. Different pulmonary diseases display varying degrees of microbiome imbalance; however, the specific variability and clinical significance of their microbiomes remain largely unexplored.

**Methods:**

In this study, we delineated the pathogen spectrum and commensal microorganisms in the lower respiratory tract of various pulmonary diseases using metagenomic sequencing. We analyzed the disparities and commonalities of the microbial features and examined their correlation with disease characteristics.

**Results:**

We observed distinct pathogen profiles and a diversity in lower airway microbiome in patients diagnosed with cancer, interstitial lung disease, bronchiectasis, common pneumonia, Nontuberculous mycobacteria (NTM) pneumonia, and severe pneumonia.

**Discussion:**

This study illustrates the utility of Metagenomic Next-generation Sequencing (mNGS) in identifying pathogens and analyzing the lower respiratory microbiome, which is important for understanding the microbiological aspect of pulmonary diseases and essential for their early and precise diagnosis.

## 1 Introduction

Serving as a respiratory organ, healthy lungs facilitate the exchange of O_2_/CO_2_ between blood and ambient air, which is crucial for sustaining the body’s normal physiological functions. Historically, the understanding of the microbiome in healthy lungs was limited, with a prevailing belief that they are sterile (Dickson et al., 2013). This belief stemmed from the lack of invasive sampling of the healthy lungs and methodological constraints, such as the difficulties to culture fastidious organisms. In 2010, Markus Hilty et al. utilized 16s rRNA sequencing to confirm the presence of bacteria in healthy lungs, identifying bacterial genera such as *Prevotella*, *Veronella*, *Streptococcus*, and *Haemophilus* in cytological brushes from the left upper lobe (~2000 bacterial genomes/cm^2^ surface area) (Hilty et al., 2010). In recent years, the advances of culture-independent molecular diagnostic methods, particularly high-throughput sequencing technologies, have significantly enhanced the identification of a broad range of pathogens in a variety of biological specimens. Studies utilizing nucleic acid sequencing of bronchoalveolar lavage fluid (BALFs) have confirmed the presence of fungi, such as *Cladosporium* and *Aspergillus* spp. and viruses, including anellovirus, Gardnerella phages and Lactobacillus phages (Wilson et al., 2019; Tian et al., 2022). Therefore, it is evident that the lung harbors a microbiome. Being connected to the external environment, human lungs experience a constant exchange of microorganisms through the upper respiratory tract (oral and nasal cavities, pharynx, and trachea) with each breath, leading to a dynamic microbial cycle and ever-changing microbiome (Natalini et al., 2023). Conversely, the microbiota absorbs nutrients and necessitates dynamic microbiological diversity, self-renewal, and maintenance from both the air phase (airways) and the fluid/cellular phase (*i.e.*, alveoli and their cellular components) within the biological niche of the lungs. The homeostasis of the pulmonary microbiome is critical for the normal physiological function of the lungs. Prior research has indicated that various pulmonary diseases, including lung cancer (Tsay et al., 2018), infectious pneumonia, interstitial pneumonia, and non-infectious diseases such as bronchiectasis (Cox et al., 2017), display alterations in the microbiome, including both pathogenic and commensal organisms of the lower respiratory tract. On the other hand, a close association was observed between pulmonary microbiome and the development and progression of various respiratory diseases, including asthma, chronic obstructive pulmonary disease (COPD), cystic fibrosis (CF), non-CF bronchiectasis, tuberculosis, COVID-19, and lung cancer (Chung, 2017; Hong et al., 2018; Ding et al., 2021; Llorens-Rico et al., 2021; Ramsheh et al., 2021). However, the similarities and differences in the microbiome across diseases and their clinical relevance remain inadequately studied.

In this study, we analyzed data from patients diagnosed with various lung diseases, admitted to the Department of Respiratory Medicine at Fujian Provincial Hospital between 2019-2022. We collected BALF, peripheral blood, and tissue samples (lung biopsies obtained from lesions), along with the conventional microbiological test results, routine clinical test results. Using mNGS on BALF, we examined the spectrum of microorganisms present in the lower respiratory tract. Meanwhile, we analyzed the microbiome composition and its association with the pulmonary disease. This study indicates that mNGS is effective in identifying pathogens and microbiome of the lower respiratory tract. Furthermore, distinct pulmonary diseases are characterized by unique pathogen and microbiome profiles. It enhances our understanding of the pathology underlying various pulmonary diseases and may benefit diagnosis and therapeutic approaches of these diseases.

## 2 Materials and methods

### 2.1 Patient enrollment

This retrospective study included 305 clinical samples from 208 patients, diagnosed with various lung diseases in the intensive care unit (ICU) of Fujian Provincial Hospital between 12 June 2019 jand 19 July 2022. The types of diseases included interstitial pneumonia, lung cancer, common or severe pneumonia, NTM pneumonia, and bronchiectasis. Data including age, sex, exposure history, comorbidities, onset, symptoms, imaging, laboratory tests, diagnostic methods, treatment, and clinical outcomes, were extracted from electronic medical records. The Ethics Committee of Fujian Provincial Hospital approved the study, and all data were anonymized before analysis. The study adhered to the Declaration of Helsinki, with data procured from the General ICU of Fujian Provincial Hospital.

### 2.2 Clinical sample collection and DNA extraction

Bronchoalveolar lavage fluid (BALF), sputum, peripheral blood and other samples were obtained from each patient, following the acquisition of consent from either the patients themselves or their surrogates. Experienced bronchoscopists collected the BALF samples after administration of anesthesia with midazolam. Peripheral blood underwent centrifugation at 1600g for 10 minutes, followed by a further centrifugation of the supernatant at 16000g for 10 minutes to isolate plasma. For other samples, genomic DNA was extracted from 1 mL of specimens. The resulting DNA underwent library preparation (enzymatic fragmentation of genomic DNA, end repairing, terminal adenylation and adaptor ligation) and purification. All steps were performed according to a previous study (Luan et al., 2021).DNA extraction and library preparation from clinical samples were conducted utilizing a point-of-care automation device (Matridx Biotechnology Co., Ltd, Hangzhou, China) (Luan et al., 2021). The quality of extracted DNAs was evaluated with a BioAnalyzer 2100 (Agilent Technologies; Santa Clara, CA, United States), in conjunction with quantitative PCR, to assess the adapters prior to sequencing. The name of the kit used for NGSmaster was Matridx Biotechnology’s Next-Generation Sequencing Reagent Kit (Cat. No. CW0531M). For DNA extraction, we used a kit from Matridx, Cat. No. MAR002 and followed SOPs provided by the manufacturer.

### 2.3 Metagenomic next-generation sequencing

Qualified DNA libraries were combined and sequenced using the Illumina NextSeq500 system (50 bp single end; San Diego, CA, United States). Each run included one negative control, consisting of artificial plasma mixed with fragmented human genomic DNA, and one positive control, comprising a mixture of inactivated bacteria, fungi, and pseudoviral particles containing synthesized DNA or RNA fragments of adenovirus and influenza A virus, for quality control. Each sample generated a total of 10 - 20 million reads. Initially, raw sequenced reads underwent quality control processing to eliminate short (length < 35 bp), low-quality, and low complexity reads, along with the adapter sequences. Sequences from the host were excluded by aligning them to the human-specific database in NCBI (GRCh38.p13), utilizing Bowtie2 (version 2.3.5.1). Clean reads were then aligned to a curated in-house microbial database, which incorporated sequences from the NCBI GenBank nucleotide (nt) database and assembly database, as well as sequences assembled from our own pure fungal cultures, with Kraken2 (version 2.1.2; confidence = 0.5) for rapid taxonomic classification. The aligned microbial reads underwent further validation through a secondary alignment to the microbial database, utilizing Bowtie2. When inconsistencies arose between the results of Kraken2 and Bowtie2, the classification of reads was determined using BLAST (version 2.9.0) (Altschul et al., 1990; Langmead and Salzberg, 2012; Wood and Salzberg, 2014). The parameters and thresholds used in BLAST included: E-value threshold: 1e-5; Identity cutoff: 90%; Alignment length cutoff: 100 base pairs. These thresholds were selected to ensure high confidence matches and to minimize false positives. Prior to data analysis, microbes identified in clinical samples were compared to those detected in NTC (no template control). Microorganisms with reads per million (RPM) above 10, or those not detected in NTC, were retained for subsequent analysis. Essentially, all microbial species were searched in PubMed to determine whether the organisms can cause pneumonia. If yes, the microorganisms were classified as pathogens.

### 2.4 Pathogen reporting criteria

Microbial reads identified from a library were reported if: 1) the sequencing data passed quality control filters (library concentration > 50 pM, Q20 > 85%, Q30 > 80%); 2) negative control (NC) in the same sequencing run does not contain the species or the RPM (sample)/RPM (NC) ≥ 5, which was determined according to previous studies as a cutoff for discriminating true-positives from background contaminations (Schlaberg et al., 2017; Wilson et al., 2019; Luan et al., 2021).

### 2.5 Statistical analysis

Categorical variables were represented as frequencies and percentages and compared utilizing Fisher’s exact test. Continuous data with normal distribution were represented as mean ± standard deviation (x ± s), while data with non-normal distribution were represented by median (range). The Wilcoxon test or Kruskal-Wallis test was employed to calculate differences and significance for non-normal distribution data. Statistical analysis was conducted using SPSS 26.0 (IBM Corporation). R (Version 4.2.1) was utilized for data visualization. Specifically, unsupervised clustering methods, referencing the core steps of limma, voom, fit, eBays were employed for bivariate or multivariate difference analysis. The limma package’s plotMDS illustrated the final similarities (or differences) between samples, and results were output through the topTable method, sorted by P-value. Before analyzing their relative abundance, RPKM values of microbes underwent log2 transformation. The limma package was utilized to analyze variations in the composition and abundance of microbes between groups. Particularly, the FDR (False Discovery Rate) method was employed to correct the primary P-value for multiple comparisons. Subsequently, corrected P-values ≤ 0.05 were deemed statistically significant.

## 3 Results

### 3.1 Microbial detection in lower respiratory tract samples from patients with different lung diseases

**Figure 1:**
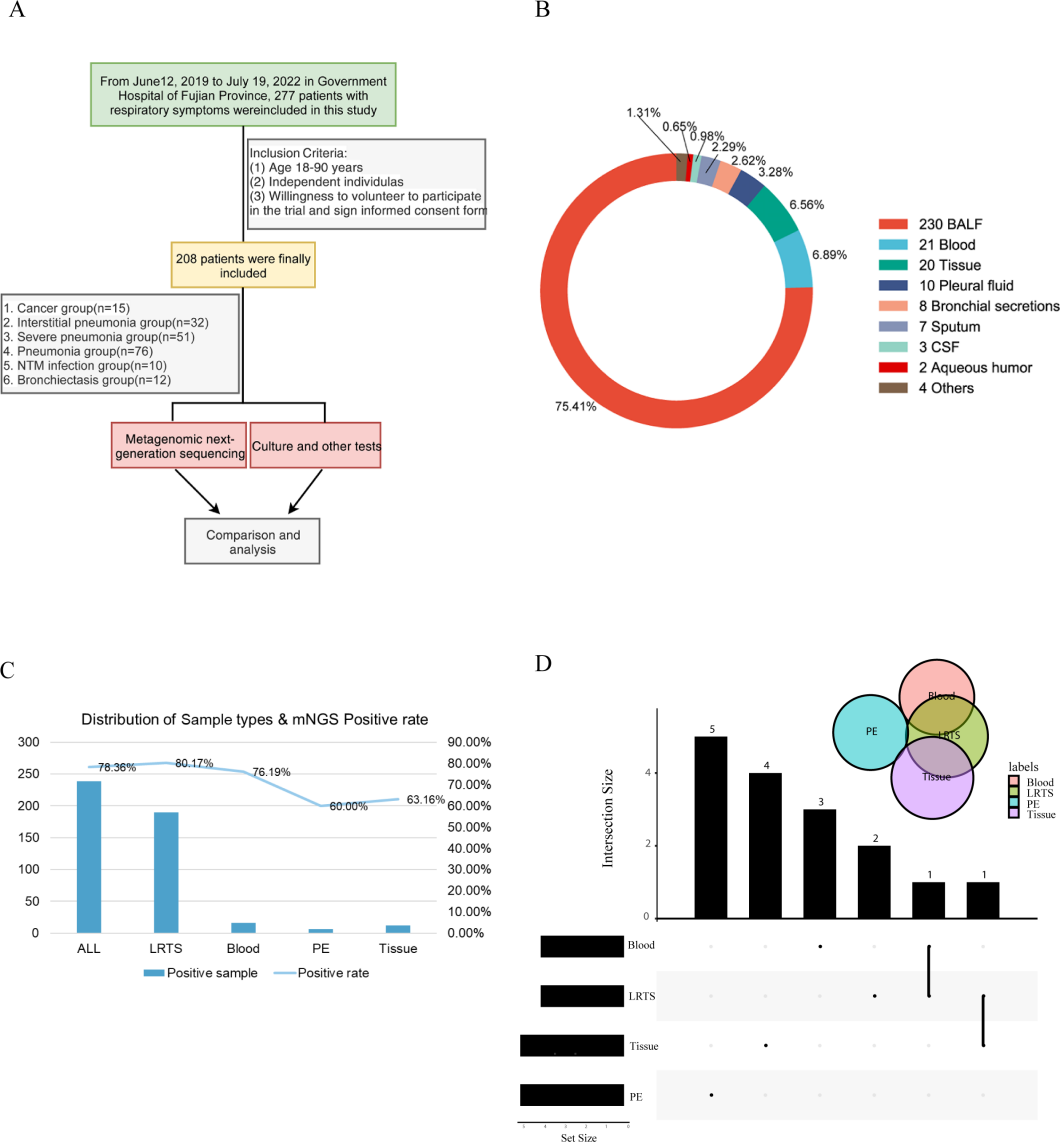
Schematic workflow of this study. **(A)** Overall research framework; **(B)** Explanation of sample types and numbers of different samples; **(C)** The number of positive samples and positivity rate of different sample types detected by Mngs. LRTS, lower respiratory tract samples; PE, pleural effusion; **(D)** Venn diagram was used to display the number of detected common and different pathogens among different sample types. The dots and connecting lines of the intersection matrix indicate the intersections between the categories, the bars on the top indicate the intersection size (number of pathogen types) of each category, and the black bars on the left of each row show the set size (number of pathogen types in total) for each sample type.

In this retrospective study, we collected data from 277 patients diagnosed with various pulmonary diseases at the Department of Respiratory Medicine of Fujian Provincial Hospital between 2019 and 2022. A total of 208 patients met the inclusion criteria. The patients were categorized into six groups based on the diagnosis: lung cancer (CA), interstitial lung disease (IP), bronchiectasis (BRO), common pneumonia (PN), NTM pneumonia (NTM), and severe pneumonia (SP) (**Figure 1A**). Of the 208 patients, 185 provided BALF samples only and 21 patients had two types of samples including BALF and others such as peripheral blood, tissue, and pleural fluid. The sample distribution of the six groups were shown in **Supplementary Figure S1**. Additionally, some patients had BALF collected at two different time points, leading to a total of 305 samples (**Figure 1B**). We also obtained results from conventional microbiological tests, routine tests, and clinical consultations.

The overall positivity rate of mNGS for pathogen detection was 78.36%, with 80.17%, 76.19%, 60.0%, and 63.16% for lower respiratory tract specimens (LRTS), peripheral blood, pleural fluid, and tissue samples, respectively (**Figure 1C**). Little overlap was observed among the pathogens identified in peripheral blood, pleural fluid, and tissue samples. In contrast, pathogens identified in BALF showed a higher degree of concordance with those in peripheral blood and tissue samples, at 28.7% and 29.6% respectively (**Figure 1D**).

**Figure 2:**
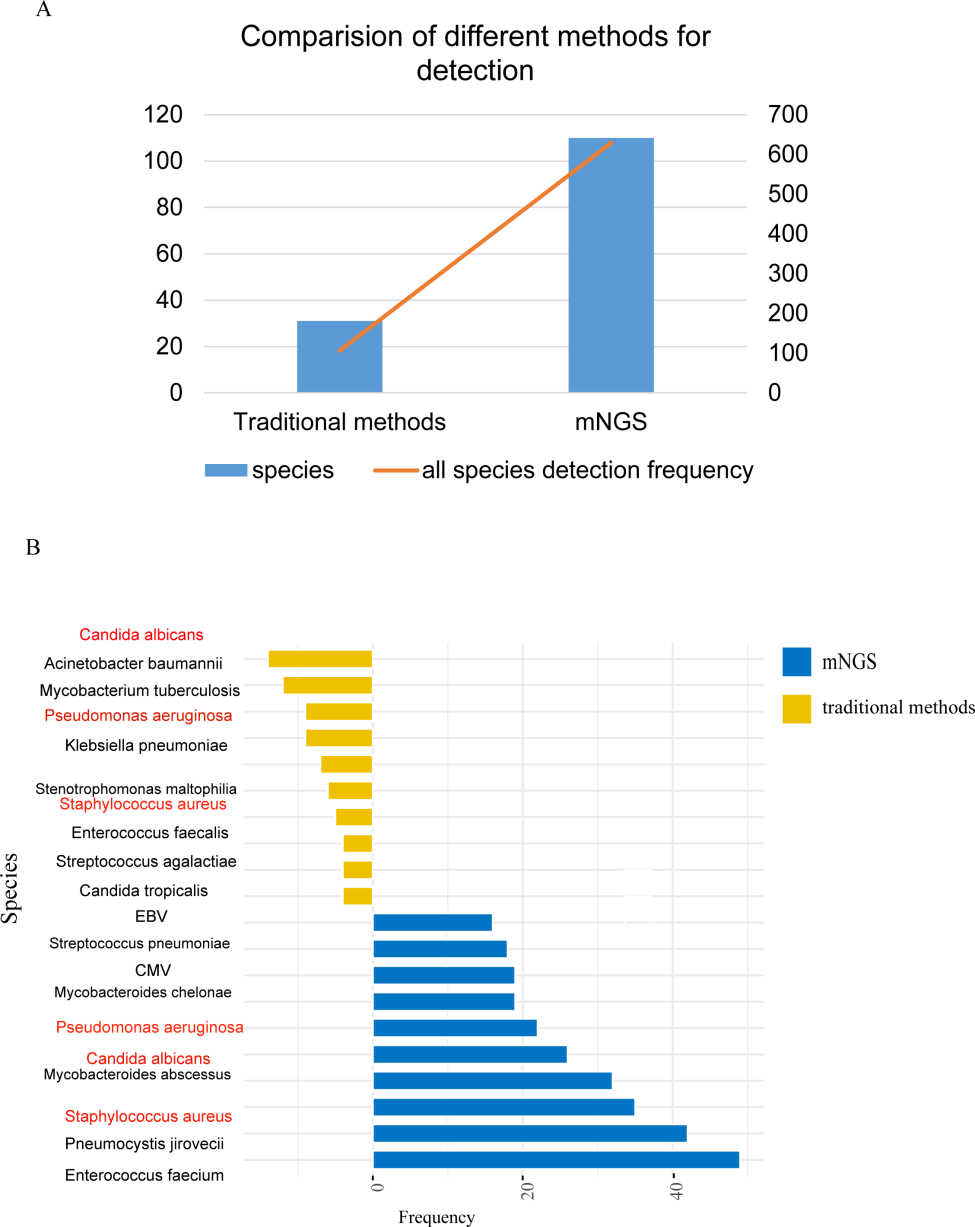
Comparison of pathogens detected by traditional pathogen detection and mNGS. **(A)** Comparison of pathogen detection numbers and detection frequencies between traditional pathogen detection methods and mNGS; **(B)** Top 10 pathogens detected by different detection methods, with red indicating pathogens detected by both methods.

The mNGS results were compared with the conventional microbiological tests (CMTs). CMTs identified 31 distinct pathogens for a total of 106 times, in contrast to mNGS that reported 110 distinct pathogens for a total of 630 times (**Figure 2A**). Subsequently, we ranked the top 10 pathogens based on frequency by both mNGS and CMTs and three species were in common: *Candida albicans*, *Pseudomonas aeruginosa*, and *Staphylococcus aureus* (**Figure 2B**).

### 3.2 The lower respiratory pathogen spectrum of different pulmonary diseases and clinical relevance

**Figure 3:**
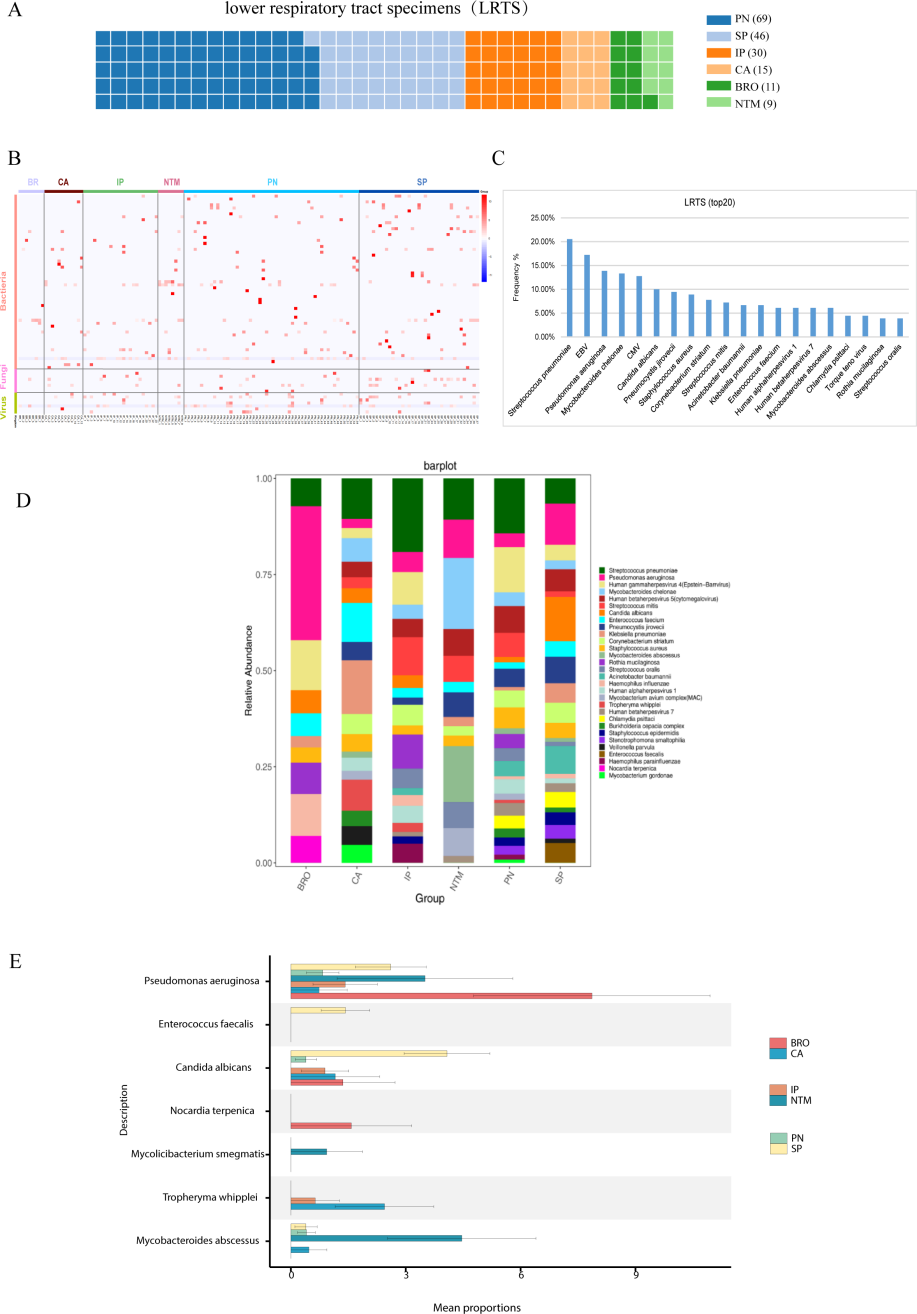
Pathogen profile analysis. **(A)** Analysis of pathogen spectrum in lower respiratory tract samples, and the contribution of lower respiratory tract samples from different groups. Each square refers to one specimen; **(B)** Pathogen distribution diagram, showing the distribution of bacteria, fungi, and viruses in the Lung cancer (CA), interstitial lung disease (IP), bronchiectasis (BRO), common pneumonia (PN), NTM pneumonia (NTM), and severe pneumonia (SP) groups; **(C)** Top 20 detected pathogens and their frequency in lower respiratory tract samples; **(D)** Pathogen stacked bar chart for different groups; **(E)** Comparison of differentially detected pathogens among groups.

The pathogen profile of different patient groups was analyzed, revealing significant disparities. For instance, the lung cancer, bronchiectasis, and NTM pneumonia groups primarily showed bacterial infections with few viruses detected. The interstitial lung disease group had bacteria, viruses, and fungi (**Figures 3A–C**). Additionally, it was observed that over 70% of the samples in each group showed mixed infections (**Supplementary Figures S2**, **S3**).

Using the Kruskal-Wallis rank sum test, we found enrichments of distinct pathogens in different groups. For instance, the bronchiectasis group showed an enrichment for Pseudomonas aeruginosa, and the detection of Tropheryma whipplei was higher in lung cancer than other groups (**Figures 3D, E**). These findings underscore the varying pathogen profiles associated with different pulmonary diseases and their clinical implications.

### 3.3 Relationship between microbiome and clinical relevance

**Figure 4:**
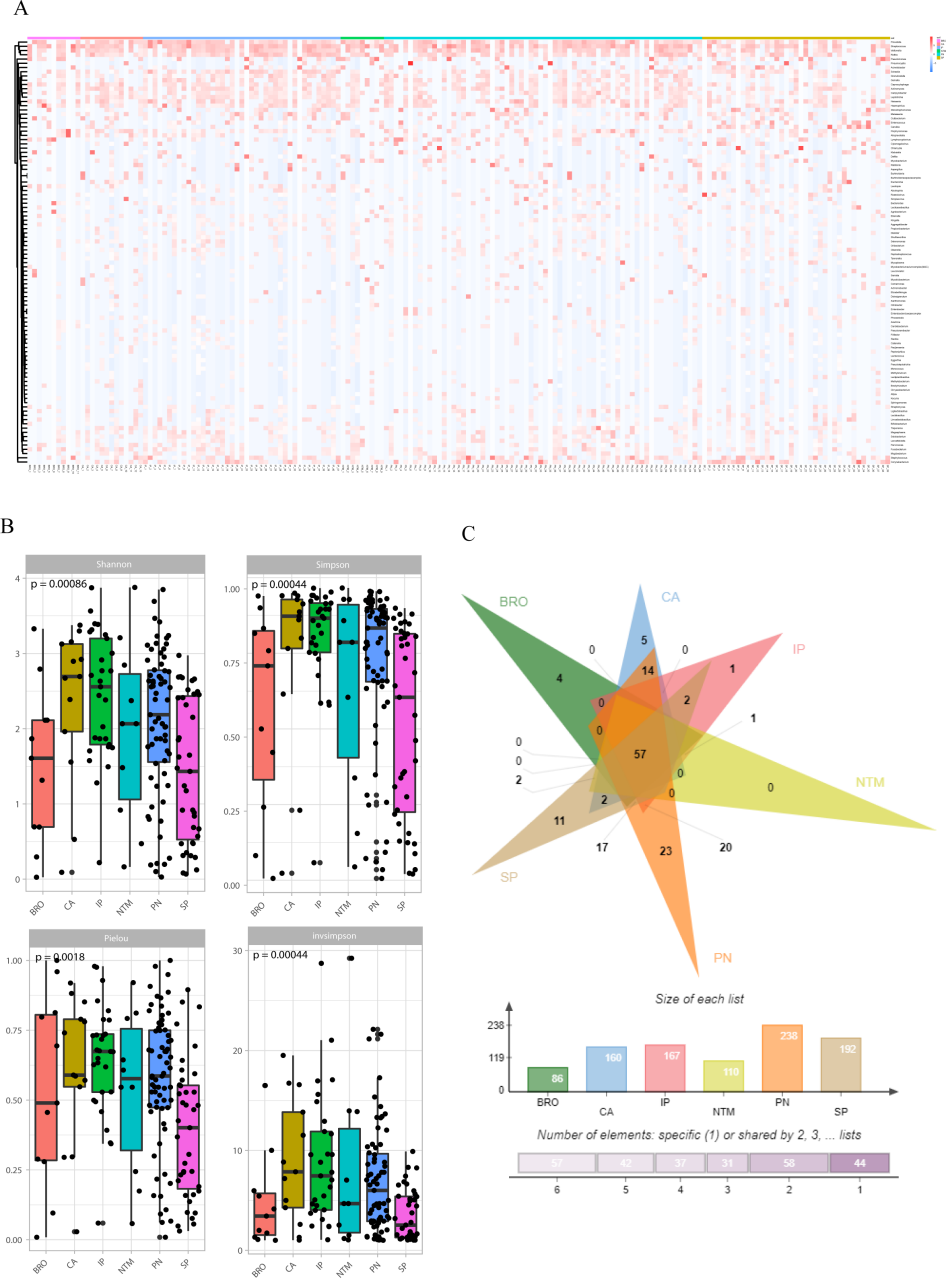
Symbiotic microbiota research. **(A)** The distribution of symbiotic microbiota in different disease groups; **(B)** Analysis of microbiota diversity, alpha diversity research; **(C)** Venn diagrams show the comparison between different groups, the top shows the overlapping situation of symbiotic microbiota between different groups, and the bottom shows the total number of symbiotic microbiota and the number of unique symbiotic microbiota for each group.

The microbiological findings of mNGS include both pathogenic and commensal microorganisms. Symbiotic microecology can potentially influence the viability of pathogenic entities and may even impact the progression or prognosis of lung diseases. Consequently, we investigated the diversity of organisms in the lower respiratory tract across various pulmonary diseases, which revealed distinct compositions and diversities of microorganisms. At the genus level, the lung cancer group was predominantly characterized by *Klebsiella* and *Pseudomonas*, the interstitial lung disease group by *Pseudomonas* and *Candida* spp. In addition, we found enrichment of *Pseudomonas* in the bronchiectasis group, *Klebsiella* and *Pseudomonas* in the common pneumonia group, *Pseudomonas*, *Klebsiella* and *Pseudomonas* in the NTM pneumonia group, *Klebsiella* and *Pseudomonas* in the severe pneumonia group (**Figure 4A**).

Alpha diversity was evaluated using four indices: Shannon (P=0.00086), Simpson (P=0.00044), Pielou (P=0.0018), and InvSimpson (P=0.00044). The results indicated significant differences in microbial diversity levels among disease groups (**Figure 4B**). As for beta-diversity, the PERMANOVA analysis indicates that there are statistically significant differences in community composition among the six patient groups with a p-value of 0.015. The R² value of 0.05451 suggests that 5.451% of the total variance is explained by differences between the groups, while 94.549% is attributed to within-group differences. The F-statistic of 1.4297 supports the presence of notable between-group differences. Despite the significant p-value, the relatively low R² value indicates that within-group variation plays a major role. This implies that while the groups differ significantly in community composition, individual variability within each group is substantial and warrants further investigation. In addition, to quantify the discriminative power of pathogen profiles among these groups, we performed a bootstrap analysis calculating the area under the curve (AUC) for each disease group. The result revealed distinct discriminatory capabilities among the groups. The ‘OTHER’ group, which amalgamated NTM pneumonia (NTM), lung cancer (CA), interstitial lung disease (IP), and bronchiectasis (BRO) due to limited sample size, exhibited an AUC of 0.8696, suggesting a high accuracy in distinguishing this composite group from others. The AUC of PN and SP groups were 0.7955 and 0.7470, respectively (**Supplementary Figure S4**). To assess the similarity and disparity of the microbiome, we enumerated the microorganisms found in different groups. The results indicated that the common pneumonia group had the highest diversity with 238 species, contrasting with the bronchiectasis group, which had the lowest (86 species). Besides the shared species, unique species were identified in each group: four in the BRO group, 11 in the SP group, 23 in the PN group, five in the CA group, and one in the IP group. Notably, no species were unique to the NTM group (**Figure 4C**).

**Figure 5:**
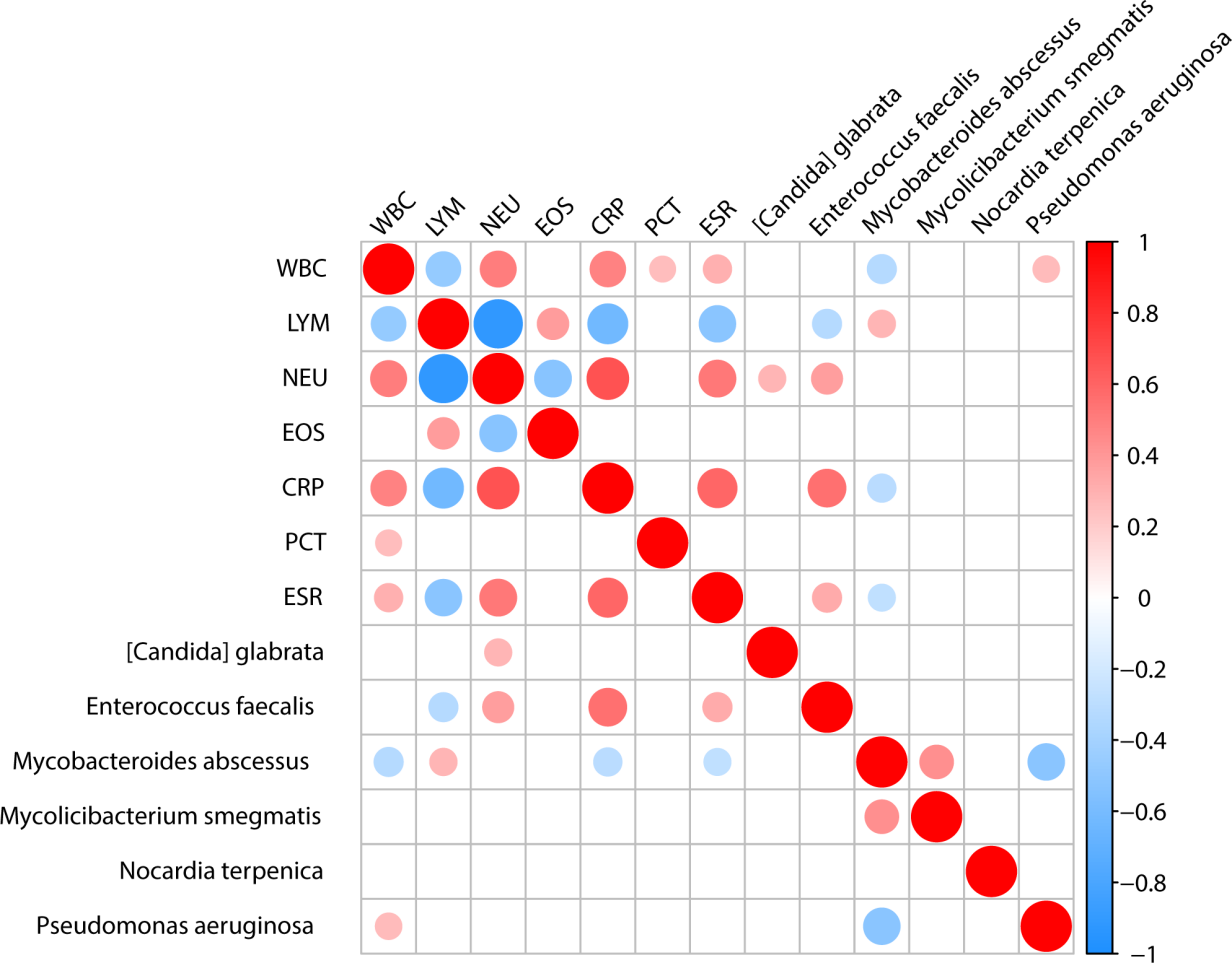
The association between clinical indicators and specific microbiota. Analysis of the correlation between important pathogens and clinical indicators shows that [Candida] glabrata is positively correlated with NEU. Enterococcus faecalis is positively correlated with NEU, CRP, and ESR clinical indicators while being negatively correlated with LYM. Mycobacteroides abscessus is positively correlated with LYM and negatively correlated with WBC, CRP and ESR. Pseudomonas aeruginosa is positively correlated with WBC.

We analyzed the correlation between microorganisms and the clinical test results, such as the biochemical test of blood. We found that *Enterococcus faecalis* showed a positive correlation with the neutrophil count, CRP, and ESR, and a negative correlation with lymphocyte count. *Mycobacteroides abscessus* showed a positive correlation with lymphocyte count and a negative correlation with white blood cell count, CRP, and ESR. *Pseudomonas aeruginosa* exhibited a positive correlation with white blood cell count (**Figure 5**).

### 3.4 Statistical analysis of age and sex in relation to disease groups

We have performed a logistic regression analysis to evaluate the relationship between age, sex, and disease groups. The results showed that the coefficient for age was -0.07600, with a p-value of 0.08353, indicating a marginal effect on disease group classification that is not statistically significant at the 0.05 level. The negative coefficient suggests a slight decrease in the log-odds of being in a specific disease group with increasing age. Conversely, the coefficient for sex was -1.54491, with a significant p-value of 0.03920, demonstrating that sex was a statistically significant predictor of disease group classification, with males having lower odds of being classified in certain disease groups compared to females. Combined with the result shown in **Table 1**, we found that males were more likely to get pneumonia and severe pneumonia than female.

**Table 1:** The patient information of different disease groups.

Group	Age (years)	Sex	Underlying disease(s)	Length of hospitalisation (Days)
Cancer	44-79	M (8), F (7)	Yes (11), No (4)	4-50
Interstitial pneumonia	38-81	M (18), F (15)	Yes (25), No (6)	4-34
Severe pneumonia	46-88	M (38), F (13)	Yes (48), No (3)	1-66
Pneumonia	14-86	M (54), F (22)	Yes (63), No (13)	2-46
NTM infection	36-82	M (5), F (5)	Yes (6), No (4)	4-15
Bronchiectasis	23-82	M (3), F (9)	Yes (8), No (4)	3-15

## 4 Discussion

Despite the similarities in clinical presentations of various pulmonary diseases, such as fever, cough, and structural changes of the lung, the underlying pathological mechanisms differ significantly (Yılmaz et al., 2017; Ren et al., 2020; Hoshina and Takei, 2021; Mathew and Mugele, 2021). To study the pathological mechanisms of different pulmonary diseases, researchers typically used *ex vivo* methodologies, including cell and molecular biology as well as *in vivo* animal models (Liao et al., 2019; Yang et al., 2021). Several studies have explored disease pathology through data mining and correlation analyses (Blanch et al., 2002; Rahman et al., 2022). Conventional microbiological tests have low detection rates, long turnaround time, and inability to identify a broad range of potential pathogens. Owing to advancements in diagnostic technology, mNGS has gained widespread acceptance for the diagnosis of infectious diseases (Fang et al., 2022). It has shown enhanced diagnostic efficacy compared to traditional methods, particularly in identifying rare or emerging pathogens. Therefore, in this study, we employed mNGS to evaluate the microbial profiles from patients with different pulmonary diseases.

Humans and microbes have coexisted in a symbiotic relationship. Termed as the second human genome, the human microbiome encompasses a diverse array of microorganisms, including bacteria, yeasts, archaea, fungi, protozoa, and viruses. These organisms and their byproducts are crucial in modulating and maturing the local microenvironments, including the immune niches of the tissues and organs, and are pivotal in maintaining physiological homeostasis and regulating the functions of organs under pathological conditions (Tsay et al., 2021). Microorganisms inhabit nearly all surfaces of the human body and the lung microbiome typically maintains a dynamic equilibrium. However, this balance is perturbed during diseases, leading to various pathological conditions and clinical manifestations, including infection and inflammation. Consequently, the imbalance and subsequent rebalancing of the lung microbiome may represent pivotal mechanisms governing the pathological conditions and progression of pulmonary diseases (Dickson et al., 2018; Tsay et al., 2018).

The respiratory tract harbors ecological niches populated by commensal and pathogenic microorganisms that are crucial for the progression of diseases (Man et al., 2017; Goeteyn et al., 2023). In the microbiota of healthy human lungs, a variety of microorganisms are typically present, and their distribution and composition can provide insights into respiratory health. In the lungs of healthy individuals, the microbial landscape is predominantly shaped by bacteria, with a few dominant phyla, such as Firmicutes (Streptococcus and Staphylococcus), Proteobacteria (Haemophilus and Pseudomonas), Bacteroidetes (Prevotella and Porphyromonas) and Actinobacteria (Corynebacterium and Mycobacterium​) (Dickson and Huffnagle, 2015). Bacteria like Streptococcus and Prevotella are often found in the lungs without causing disease, playing roles in maintaining the microbial balance and potentially stimulating the immune system​, and Organisms like Pseudomonas aeruginosa and certain Staphylococcus species, while sometimes part of the normal microbiota, can become pathogenic under certain conditions, such as in individuals with compromised immune systems or underlying lung diseases​ (Dickson and Huffnagle, 2015).

The microbial diversity within the lungs can be modulated by various biotic and abiotic factors (Liu et al., 2022). An imbalance in the respiratory microbiome can facilitate colonization by opportunistic pathogens, culminating in respiratory infections, including pneumonia (Wypych et al., 2019; Hernández-Terán et al., 2021). Alterations in the microbiome are observed during infections of the lower respiratory tract and are closely correlated with the course and prognosis of pneumonia (Gu et al., 2019). Hence, a deeper understanding of alterations in microbiome composition is important for elucidating the role of pathogens in pulmonary infections. Studies have uncovered the microbial composition of the lungs in patients with bacterial meningitis (Moon et al., 2019), refractory *Mycoplasma pneumoniae* pneumonia (Shi et al., 2022; Deng et al., 2023), pulmonary tuberculosis (Chao et al., 2021; Zhang et al., 2023), and invasive pulmonary aspergillosis (Hérivaux et al., 2022) using untargeted pathogen metagenomics or 16S rRNA gene sequencing. While 16S rRNA sequencing can identify bacterial species, it lacks the resolution offered by metagenomic technologies, such as shotgun sequencing, particularly in closely related species (Gupta et al., 2019).

Distinct pulmonary diseases exhibited varying microbiological features; for instance, we found microbial compositions of the lower respiratory tract of patients having lung cancer, bronchiectasis, and NTM pneumonia primarily consisted of bacteria. This is consistent with the understanding that bacterial communities can influence or be influenced by the pathophysiology of chronic respiratory diseases and cancer. For interstitial lung disease, common and severe pneumonia, these conditions had a more diverse microbial composition, including significant presences of fungi and viruses. This suggests a complex interplay in these diseases, potentially implicating more varied and severe pathogenic processes. Different types of disease showed enrichment of microorganisms, exhibiting a correlation with the clinical manifestations of each disease. For instance, the enrichment of certain bacteria in lung cancer could relate to the inflammatory and tumor-promoting environment, whereas the presence of fungi and viruses in interstitial lung disease could be linked to the disease’s multifactorial etiology involving both immune response and environmental exposures.

**Figures 3D, E** illustrate the distribution of various bacteria across different pulmonary diseases, highlighting their potential roles in these conditions. Notably, Streptococcus pneumoniae appears frequently in a variety of lung diseases, including pneumonia, supporting its status as a primary pathogen (Shoar and Musher, 2020)​. The data in these charts align with research on the role of the microbiome in respiratory health, suggesting a potential causative relationship between specific microbial communities and diseases​ (Dietl et al., 2021)​. Additionally, **Figures 3D, E** highlight the significant presence of *Pseudomonas aeruginosa* in various pulmonary diseases, correlating with its known impact on chronic conditions like cystic fibrosis and non-CF bronchiectasis. The data from these charts show its prevalence and distribution across disease groups, underlining the challenges in managing this pathogen due to its resistance to multiple antibiotics. These findings emphasize the need for targeted antibiotic therapies to improve patient outcomes by effectively managing *Pseudomonas aeruginosa* infections, supporting ongoing research into tailored treatment strategies​ (Reynolds and Kollef, 2021; Eklof et al., 2024)​.

The diversity of microbiome in the lower respiratory tract varied among disease groups, with the highest diversity observed in the lung cancer and interstitial lung disease groups, succeeded by the common pneumonia and NTM pneumonia groups, and the lowest diversity in the bronchiectasis and severe pneumonia groups. High microbial diversity in lung cancer and interstitial lung disease groups could be linked to the chronic and progressive nature of these diseases, where a diverse microbial environment might influence disease progression and response to treatment. Common pneumonia and NTM pneumonia exhibited moderate diversity, this intermediate level might reflect the specific pathogenic involvement and the body’s immune response to these conditions. Low diversity in severe pneumonia might indicate a dominance by pathogenic microbes that outcompete other members of the microbiota during acute disease phases. In bronchiectasis, reduced diversity could be associated with chronic infection or inflammation driven by a limited range of pathogens.

The AUC values obtained from our ROC analysis provide valuable insights into the performance of our classification models. The Combine group, with an AUC of 0.8696, demonstrates robust classification ability, indicating a strong relationship between disease types and pathogen abundance within this group. This suggests that the combined dataset effectively captures relevant features, leading to better discrimination between classes. In contrast, the PN and SP groups exhibit lower AUC values of 0.7955 and 0.7470, respectively, revealing comparatively weaker model performance. These differences may highlight variations in how well the pathogen abundance correlates with disease types across different datasets or groups. The observed AUC values support the hypothesis that microbial ecology could be closely related to disease states, with the Combine group showing a more pronounced relationship. This aligns with the notion that a comprehensive dataset might enhance our ability to uncover associations between microbial communities and diseases. However, the lower AUCs in PN and SP groups suggest that additional factors or more nuanced features may be needed to improve classification performance in these contexts. Further investigation into these groups could reveal underlying complexities in the relationship between pathogen abundance and disease.

This study illustrates the use of mNGS in identifying pathogens and analyzing the lower respiratory microbiome. Our findings indicate that distinct pulmonary diseases show unique microbial features, which may be linked to the pathophysiology of different diseases.

## Data Availability

The datasets presented in this study can be found in online repositories. The names of the repository/repositories and accession number(s) can be found in the article/**Supplementary Material**.
