# Supplementary material for: Pathogen spectrum and microbiome in lower respiratory tract of patients with different pulmonary diseases based on metagenomic next-generation sequencing
Source: Front Cell Infect Microbiol. 2024 Oct 31;14:1320831. doi: 10.3389/fcimb.2024.1320831 (PMC11560916; doi:10.3389/fcimb.2024.1320831)
Supplement: Supplementary file 1 [file DataSheet1.docx]

Supplementary Material

**Pathogen Spectrum and Microbiome in Lower Respiratory Tract of Patients with Different Pulmonary Diseases Based on Metagenomic Next-Generation Sequencing**

**Rujun Hong^1^**^†^**, Sheng Lin^1^**^†^**, Siting Zhang^1^**^†^**, Yaxing Yi^2^**^†^**, Lanfeng Li^1^, Haitao Yang^1^, Zhenshan Du^2^, Xuefang Cao^2^, Wenjie Wu^2^*, Ruotong Ren^2,3^*, Xiujuan Yao^1^*, Baosong Xie^1^***

*** Correspondence:** Baosong Xie: [xbaosong@126.com](mailto:xbaosong@126.com), Xiujuan Yao: [953381422@qq.com](mailto:953381422@qq.com), Ruotong Ren: [ruorabbit@163.com](mailto:ruorabbit@163.com); Wenjie Wu: jiezi_1@126.com

# Supplementary Figures

#
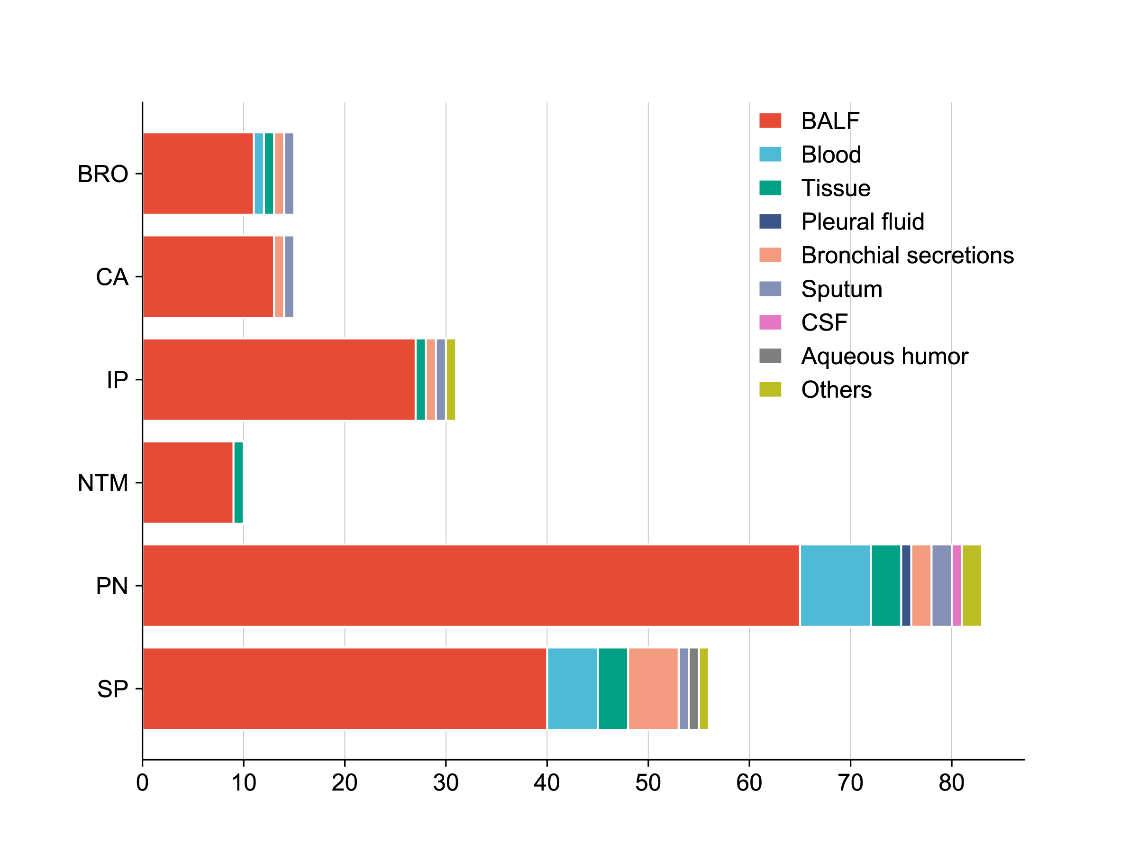


**Supplementary Figure 1.** The composition of the sample groups in different disease groups. CA, cancer group; IP, Interstitial pneumonia group; SP, Severe pneumonia group; PN, Pneumonia group; NTM, Nontuberculosis mycobacteria infection group; BRO, Bronchiectasis group; BALF, Bronchoalveolar lavage fluid; CSF, Cerebrospinal fluid.


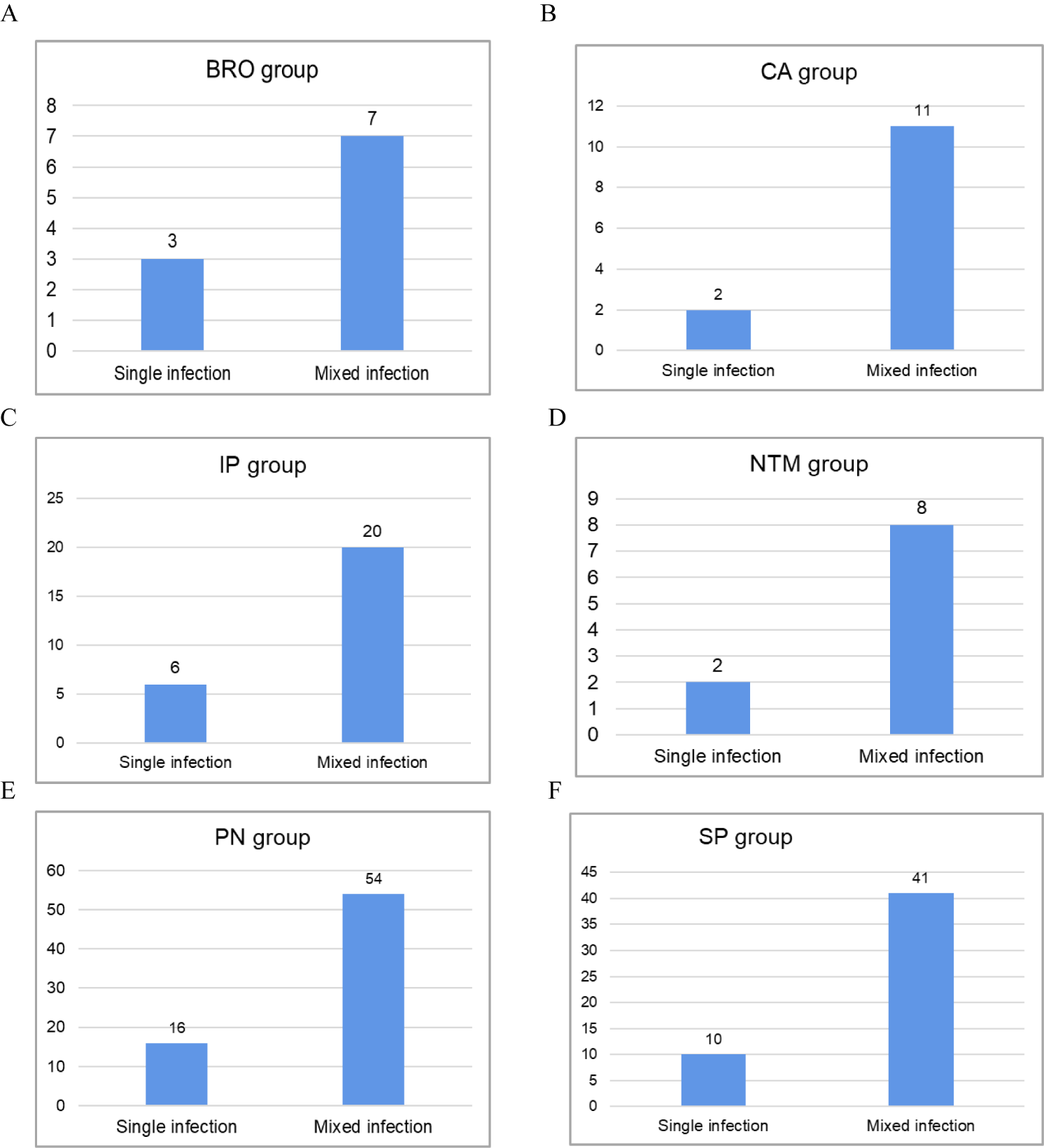


**Supplementary Figure 2.** Infection status of samples from different disease groups.


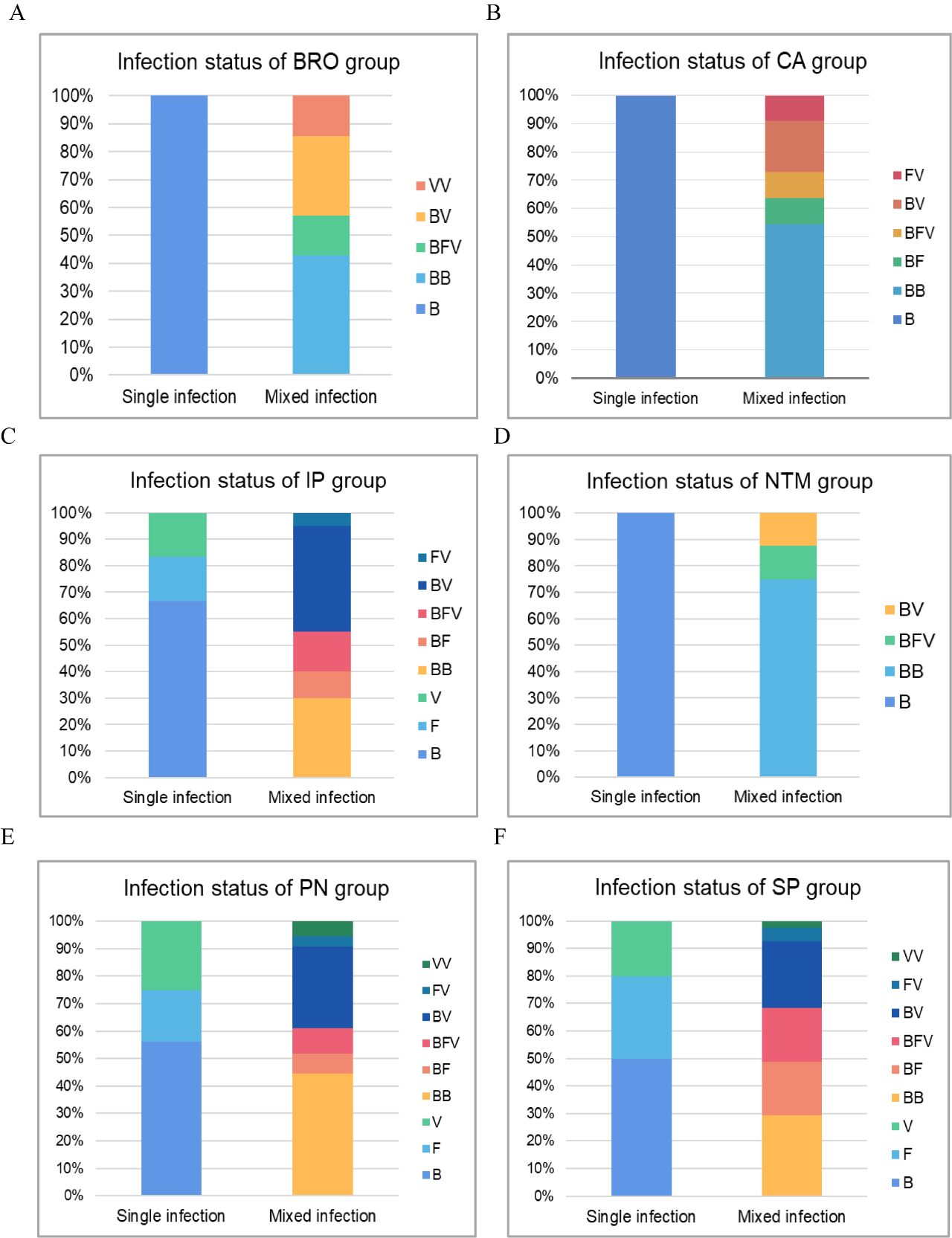


**Supplementary Figure 3.** Differentiation of single and co-infections in samples from different disease groups. VV, Viral coinfection; FV, Fungal-viral coinfection; BV, Bacterial-viral coinfection; BFV, Bacterial-fungal-viral coinfection; BF, Bacterial-fungal coinfection; BB, Bacterial-Bacterial coinfection; V, viral single infection; F, Fungal single infection; B, Bacterial single infection.


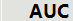

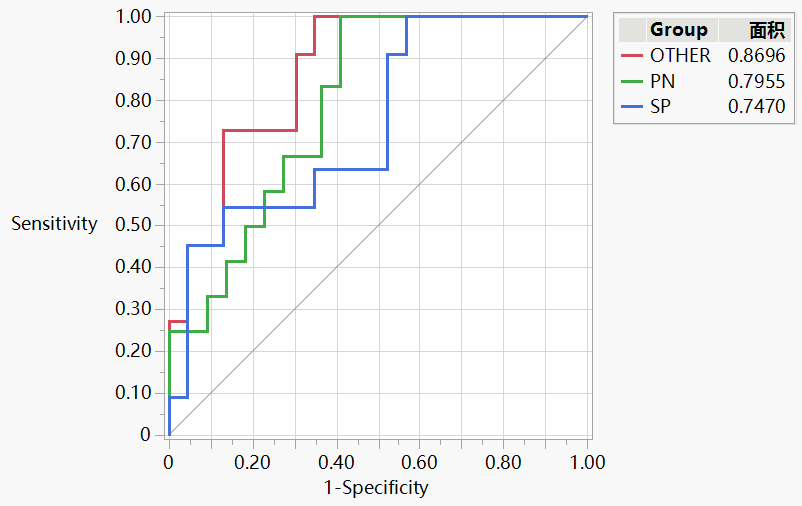


**Supplementary Figure 4.** Bootstrap analysis of different clinical groups, including common pneumonia (PN), severe pneumonia, (SP) groups and OTHER (including NTM pneumonia (NTM), and Lung cancer (CA), interstitial lung disease (IP), bronchiectasis (BRO)).
